# Analysis of PSMA expression and outcome in patients with advanced Prostate Cancer receiving ^177^Lu-PSMA-617 Radioligand Therapy

**DOI:** 10.7150/thno.47251

**Published:** 2020-06-19

**Authors:** Robert Seifert, Konstantin Seitzer, Ken Herrmann, Katharina Kessel, Michael Schäfers, Jens Kleesiek, Matthias Weckesser, Martin Boegemann, Kambiz Rahbar

**Affiliations:** 1 Department of Nuclear Medicine, University Hospital Münster, Münster, Germany.; 2 Department of Nuclear Medicine, University Hospital Essen, Essen, Germany.; 3 German Cancer Consortium (DKTK).; 4 West German Cancer Center.; 5 Department of Urology, University Hospital Münster, Münster, Germany.; 6 Division of Radiology, German Cancer Research Center, Heidelberg, Germany.

**Keywords:** PSMA radioligand therapy, PSMA PET, prostate cancer, prognosticator

## Abstract

**Rationale:** PSMA-PET-CT enables measuring molecular expression of prostate-specific membrane antigen (PSMA) *in vivo*, which is the target molecule of ^177^Lu-PSMA-617 (Lu-PSMA) therapy. However, the correlation of PSMA expression and overall survival (OS) in patients treated with Lu-PSMA therapy is currently unclear; especially with regard to coexistence of high and low PSMA expressing metastases. To this end, this retrospective single arm study elucidates the correlation of PSMA expression and overall survival in patients treated with Lu-PSMA therapy. Additionally, PET based criteria to define low PSMA expression were explored.

**Methods:** Eighty-five patients referred to Lu-PSMA therapy were included in the analysis. Pretherapeutic ^68^Ga-PSMA-PET-CT scans were available for all patients. SUV_max_ of the highest PSMA expressing metastasis (PSMA_max_), SUV_max_ of the lowest PSMA expressing metastasis (PSMA_min_), and average SUV_max_ of all metastases (PSMA_average_) amongst other PET parameters were measured for each patient. A log-rank cutoff-finder was used to determine low (lowPSMA_average_) and high (highPSMA_average_) average PSMA expression as well as low (lowPSMA_min_) and high (highPSMA_min_) minimal PSMA expression.

**Results:** PSMA_average_ was a significant prognosticator of overall survival in contrast to PSMA_max_ (HR: 0.959; p = 0.047 vs. HR: 0.992; p = 0.231). Optimal log rank cut-offs were: PSMA_average_ = 14.3; PSMA_min_ = 10.2. Patients with low average PSMA expression (lowPSMA_average_) had significantly shorter survival compared to those with high average expression (highPSMA_average_) (5.3 vs. 15.1 months; p < 0.001; HR: 3.738, 95%CI = 1.953-7.154; p < 0.001). Patients with low PSMA expressing metastases (lowPSMA_min_) had shorter survival compared to those without a low PSMA expressing metastasis (highPSMA_min_) (p = 0.003; 7.9 months vs. 21.3; HR: 4.303, 95%CI = 1.521-12.178; p = 0.006). Patients that were classified as highPSMA_average_ but with lowPSMA_min_ had an intermediate overall survival (11.4 months; longer compared to lowPSMA_average_, 5.3 months, p = 0.002; but shorter compared to highPSMA_min_, 21.3 months, p = 0.02).

**Conclusion:** Low average PSMA expression is a negative prognosticator of overall survival. Absence of low PSMA expressing metastases is associated with best overall survival and the maximum PSMA expression seems not suited to prognosticate overall survival. Low PSMA expression might therefore be a negative prognosticator for the outcome of patients treated with Lu-PSMA therapy. Future studies are warranted to elucidate the degree of low PSMA expression tolerable for Lu-PSMA therapy.

## Introduction

There are only limited therapeutic options for patients with metastatic castration-resistant prostate cancer (mCRPC) 1. However, the treatment of mCRPC patients with ^177^Lu-PSMA-617 (Lu-PSMA) achieves biochemical response (> 50% decline of prostate-specific antigen blood levels) in 45-64% of patients 2-4. Yet, the identification of mCRPC patients who will benefit from Lu-PSMA therapy is still an unmet clinical issue 5,6.

Prostate-specific membrane antigen (PSMA) targeted positron emission tomography computed tomography (PET-CT) can visualize the target molecule of Lu-PSMA therapy *in vivo*, which is also referred to as theranostics 6-9. The molecular expression of PSMA should be directly linked to Lu-PSMA efficacy. Therefore, procedure guidelines of the European Association of Nuclear Medicine (EANM) for Lu-PSMA therapy demand a PSMA-PET-CT acquisition to evaluate therapy eligibility 10. However, there are contradictory reports on the implications of PSMA targeted imaging: It has been reported that PSMA-PET is not suited to predict response to Lu-PSMA therapy 11. On the other hand, high tumor uptake in post Lu-PSMA therapy scintigraphies is a prognosticator of survival 12. It remains currently unclear to what extent PSMA expression measured by PSMA-PET-CT can predict response and prognosticate overall survival and thus, ultimately assess eligibility for Lu-PSMA therapy. Moreover, there is no reasonable definition of low PSMA expression.

The first prospective Phase II trial by Hofman et al. has addressed the issue of eligibility assessment pragmatically by requesting an arbitrarily defined minimum PSMA-PET uptake of metastases to undergo Lu-PSMA therapy 3,13. The minimum PSMA uptake of any metastasis was defined as 1.5 times the mean liver uptake 3. Only patients whose SUV_max_ exceeded this minimum activity at any metastatic site were eligible for Lu-PSMA therapy. It seems plausible that PSMA-PET uptake should be linked to therapeutic efficacy of Lu-PSMA therapy. However, it appears difficult to translate the trial inclusion criteria to the clinical routine, as it was not part of the study evaluation. By applying a SUV_max_ based criterion, Lu-PSMA therapy might be withheld from patients due to their low PSMA expression that still might have benefited from therapy. Therefore, the aim of the present study was to investigate the relevance of PSMA-PET parameters for the overall survival of patients treated by Lu-PSMA therapy. Additionally, multiple PSMA-PET parameters were employed to distinguish between patients with low and high PSMA expression. Finally, survival time, presence of liver metastases and history of second line chemotherapy were compared between patients with low and high PSMA expression.

## Methods

### Patients

All patients who were referred for ^177^Lu-PSMA-617 therapy at the Department of Nuclear Medicine in Muenster between December 2014 and October 2018 were considered in this retrospective analysis. Inclusion criterion was the presence of a ^68^Ga-PSMA-11 PSMA-PET-CT examination prior to administration of the first therapy cycle showing any uptake of the tracer in the target lesions. The decision for Lu-PSMA-617 therapy was made by the institutional interdisciplinary tumor board on a case by case basis. Prerequisites for ^177^Lu-PSMA-617 therapy were: castration-resistance (mCRPC), sustained androgen deprivation therapy, if no contraindication was present at least one line of taxane chemotherapy, PSMA-positive metastases, sufficient hematological reserve, and sufficient kidney as well as liver function 10. Pretherapeutic PSMA-PETs were assessed visually for presence of PSMA positive metastases; no quantitative PSMA-PET related inclusion criteria were applied. Foci that were not caused by physiological uptake and showed higher activity than the surrounding tissue were assessed as sufficient for Lu-PSMA therapy. A detailed patient characteristic is given by *Table 1.*

### PSMA-PET imaging procedure

^68^Ga-PSMA-11 was produced according to manufactures recommendations (precursor delivered by ABX GmbH, Radeberg, Germany). A Siemens Biograph mCT (Siemens Healthineers, Knoxville, TN, United States) was used for image acquisition. PET-CT acquisitions were started 60 minutes after tracer injection. Low-dose or diagnostic CT were acquired immediately prior to PET acquisition for anatomical orientation and attenuation correction. PET reconstruction was done using the standard software as provided by the manufacturer and an iterative time-of-flight algorithm without PSF correction. The median interval between PET acquisition and therapy start was 32 (IQR: 22) days.

### PSMA therapy preparation and administration

^177^Lu-PSMA-617 was prepared as described elsewhere (Lutetium: ITG Isotopes Technology, Garching, Germany; precursor: ABX advanced biochemical compounds, Radeberg, Germany) 14. Lu-PSMA was administered every 8 weeks until severe adverse reactions, altered therapy regime, progression, or death occurred.

### PSMA-PET image analysis

The analysis of PSMA-PET images was done semi-automatically using the research prototype software MI Whole Body Analysis Suite (MIWBAS, v1.0, Siemens Medical Solutions USA, Inc., Knoxville, TN), which has been described in detail before 15. Briefly, all PSMA avid foci were automatically pre-selected based by a pre-defined threshold; foci with a PET volume smaller than 0.5 ml (segmented by 50% of local SUVmax as threshold) were discarded. Missed pathological foci were manually added, if necessary. PSMA avid foci that were caused by physiological tracer accumulation were semi automatically removed from the analysis.

All PSMA avid metastases (regardless of SUV_max_) were segmented and SUV_max_, SUV_mean_, SUV_peak_ were reported for each metastasis. Metastases were delineated using relative thresholding (50% of local SUV_max_). On a per patient level, the mean of all SUVmax measurements (PSMA_average_), the maximum SUVmax measurement (PSMA_max_), the lowest SUV_max_ measurement (PSMA_min_) and the standard deviation of the SUV_max_ measurements (PSMA_std_) were noted.

PSMA measurements were analyzed as continuous parameter and in binarized form. When PSMA_average_ was binarized using optimized log rank thresholds, the group with low PSMA_average_ was denoted lowPSMA_average_ (highPSMA_average_; threshold: 14.3). When PSMA_min_ was binarized, patients with low PSMA expressing metastases were denoted lowPSMA_min_ (without low PSMA expressing metastases: highPSMA_min_; threshold: 10.2). The volumetric fraction of low PSMA expressing tumor was determined by dividing the volume of metastases with low PSMA expression (SUV_max_ ≤ 10.2) by the whole-body tumor volume.

### Statistical analysis

SPSS 25 (IBM, NY, USA) was used for log rank tests, Pearson correlation, Mann-Whitney-U test, Fisher's exact test and uni- as well as multivariate Cox-regression. R and the maxstat package were used for finding the optimal log rank cut-off of continuous variables 16,17. P values < 0.05 were regarded as statistically significant. To correct for log-rank test alpha error accumulation, significance was assumed when p < 0.0125 (Bonferroni correction for 4 SUV_max_ tests: optimal log rank cut-off for PSMA_max_, PSMA_min_, PSMA_average_, PSMA_std_. Other SUV parameters (SUV_mean_, SUV_peak_) were only analyzed to further corroborate SUV_max_ findings and therefore not regarded for Bonferroni correction. Values are presented together with the interquartile range (IQR).

## Results

### PSMA therapy and patient characteristics

A detailed patient characteristic is given by *Table 1.* Median therapy interval (including therapy pauses) was 8.2 (IQR: 3.3) weeks, median therapeutic activity was 6.2 (IQR: 1.2) GBq. The median cumulated dose was 23.7 (IQR: 25.7) GBq. Eighty percent of all patients had received taxane based chemotherapy (Docetaxel or Cabacitaxel), whereas 100% patients had received androgen deprivation therapy and 97.7% had received next generation androgen receptor targeted therapy (Enzalutamide or Abiraterone).

### Descriptive statistics of baseline PET parameter measurements

The median of PSMA_max_ measurements was 44.6 SUV (range 7.1-181.6), whereas the median of PSMA_average_ measurements was 18.9 (range 4.6-129.8). The median intensity was 31.6 (range 4.7 -159.7) for the highest SUV_peak_. A detailed report on SUV parameters is provided by *Table 2*.

### Baseline PET-parameters and overall survival

**Figure 1:**
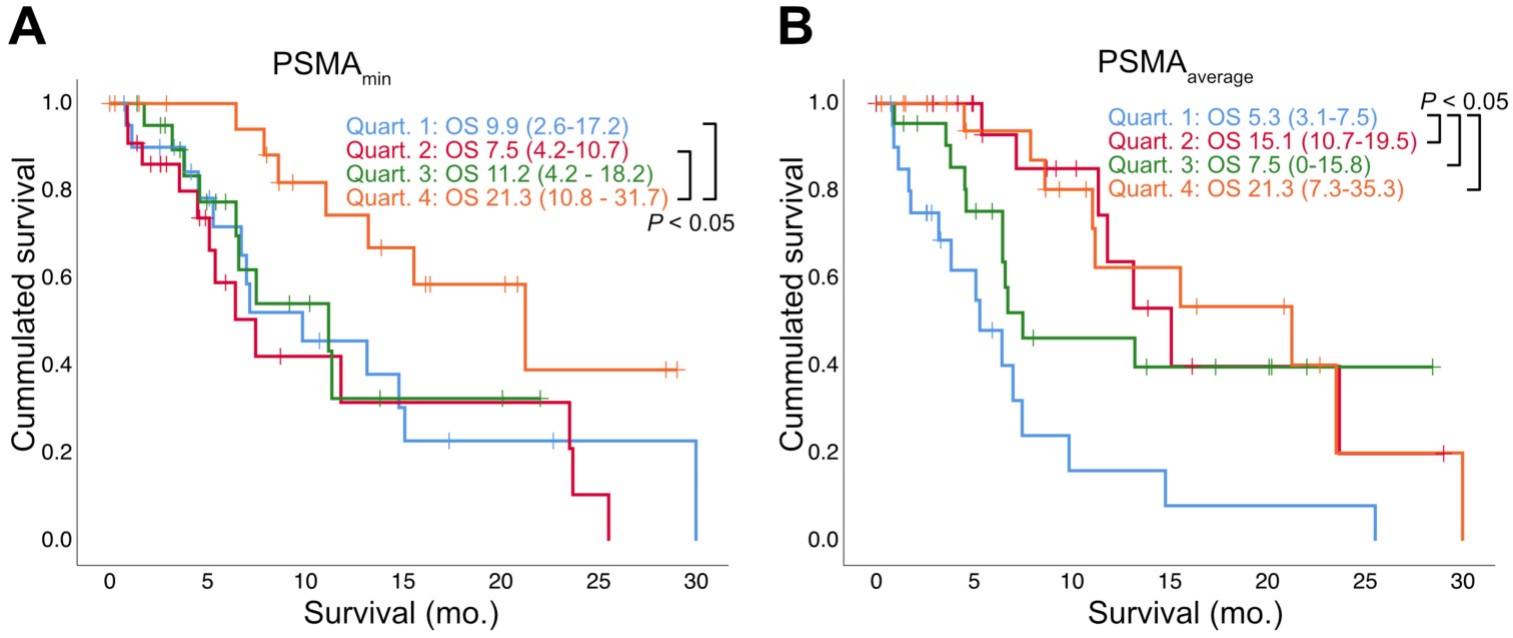
** Survival stratified by quartiles of PSMA-PET parameters.** Estimated median overall survival (OS) in months (mo.) is shown together with the 95% Confidence Interval (in parentheses). Log rank test was used to compare OS between quartiles.

Regarding SUV_max,_ neither the highest (PSMA_max_: HR: 0.992; p = 0.231; 95% CI: 0.979-1.005), nor the lowest (PSMA_min_: HR: 0.890; p = 0.118; 95% CI: 0.768-1.030) value per patient were significant prognosticators of overall survival, but the average value was (PSMA_average_: HR: 0.959; p = 0.047; 95% CI: 0.921-0.999). The same was true for SUV_mean_ (highest: HR= 0.989; p = 0.241; 95% CI= 0.970-1.008; average: HR = 0.941; p = 0.045; 95% CI= 0.887-0.999; lowest: HR = 0.799; p = 0.052; 95% CI = 0.638-1.002). Details (including the standard deviation of SUV_max_, SUV_mean_ and SUV_peak_) are given by *Table 3*. *Figure 1* depicts the overall survival stratified according to the quartiles of PSMA_average_ and PSMA_min_.

**Figure 2:**
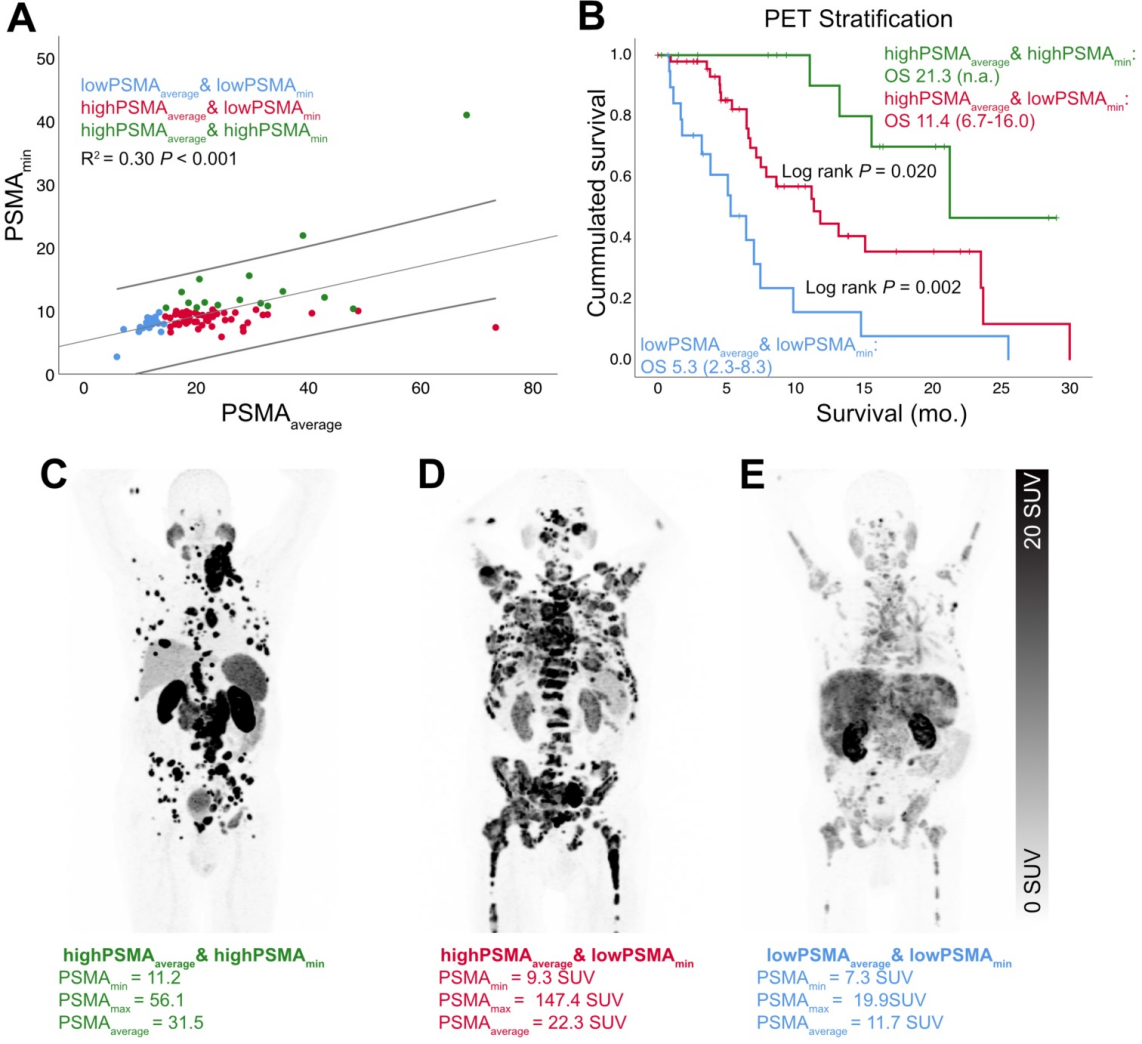
** Survival stratified by high, intermediate and low PSMA expression.** There was no relevant correlation between PSMA_min_ and PSMA_average_ (A, linear regression and 95% CI interval). Therefore, the combination of both PET parameters enabled an optimized stratification according to overall survival (B). Exemplary patients of the high (C), intermediate (D) and low (E) overall survival group were shown additionally.

**Figure 3:**
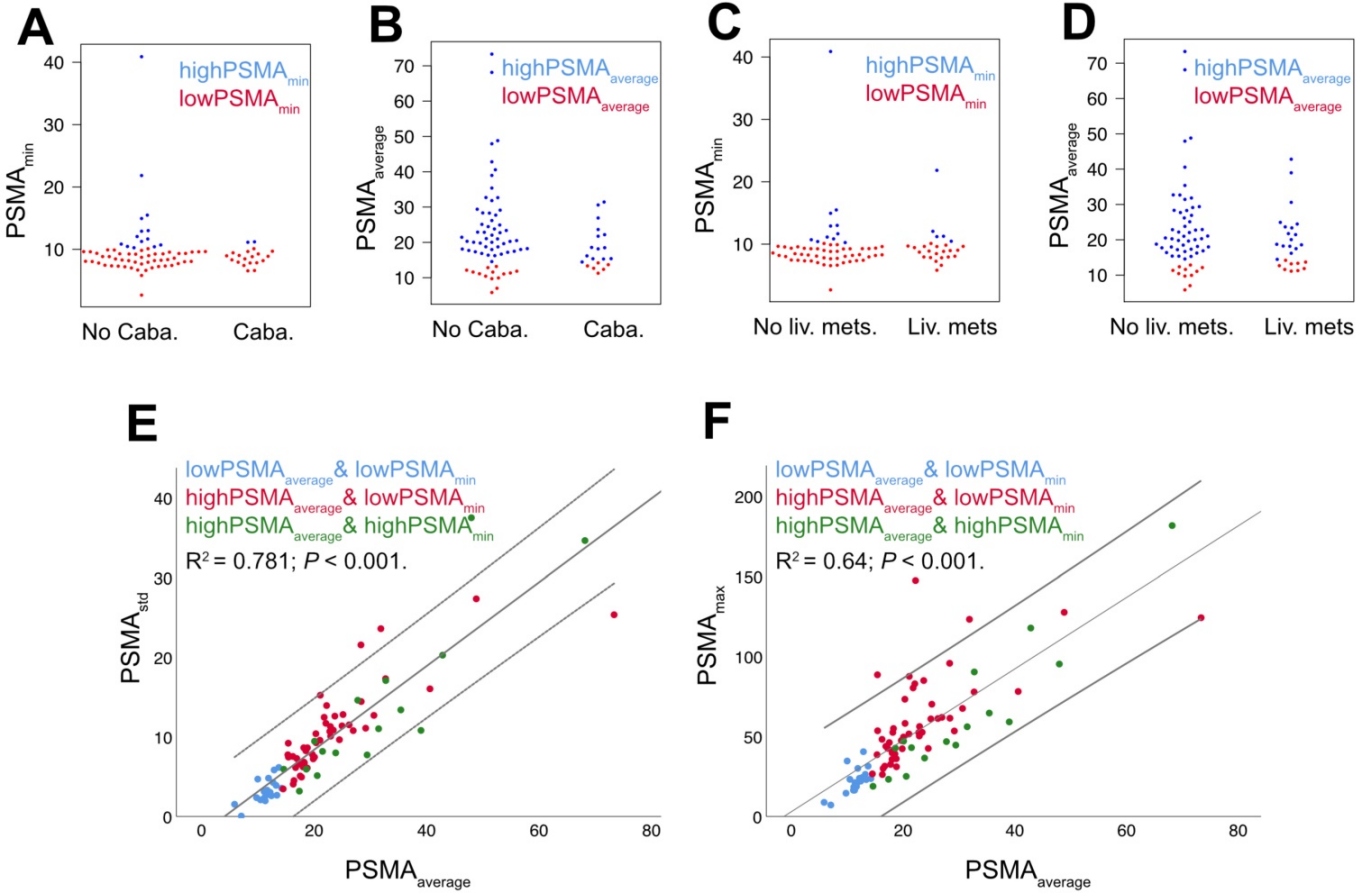
** Relations of baseline PSMA-PET parameters.** PSMA-PET parameters were grouped by history of Cabazitaxel chemotherapy (= Caba; A+B) or presence of liver metastases (= Liv. mets; C+D); there were no statistically significant differences. There was a high correlation of PSMA_std_ and PSMA_average_ and a moderate correlation of PSMA_max_ and PSMA_average_ (E+F, linear regression and 95% CI interval).

There were no relevant correlations between PSMA_min_ and PSMA_average_ (R^2^ = 0.30, p < 0.001; *Figure 2*) or PSMA_min_ and PSMA_std_ (R^2^ = 0.18, p < 0.001), but PSMA_std_ and PSMA_average_ were significantly correlated (R^2^ = 0.78, p < 0.001; *Figure 3*).

### Low PSMA expression and overall survival

In a first approach, a liver specific SUV threshold was used (1.5 × SUV*mean* liver) in analogy to Hofman et al. In our cohort, zero patients had a SUV_max_ below the liver specific threshold. Patients (n=8) with at least one tumor lesion below the liver specific SUV threshold did not have a significantly shorter overall survival time (log rank: p = 0.335; 7.5 vs.13.2 months; HR: 1.588; p = 0.340; 95% CI: 0.615-4.101).

In a second approach, PSMA_average_ and PSMA_min_ were binarized to determine low and high PSMA expression. The optimized log rank threshold for PSMA_average_ to stratify according to overall survival was 14.3 SUV (log rank: p < 0.001; estimated median: 15.1 vs. 5.3 months; high PSMA_average_: HR = 0.268, 95%CI = 0.140-0.512, p<0.001) and 10.2 for PSMA_min_ (log rank: p = 0.003; estimated median: 21.3 vs. 7.9 months; high PSMA_min_: HR = 0.232, 95%CI = 0.082-0.658; p = 0.006). Taken together, both classifiers stratified patients into high, intermediate or low overall survival. *Table 4* presents the intersection of these two classifiers*; Figure 2* depicts the overall survival according to them. Patients that were classified highPSMA_min_ had longer survival compared to patients classified as both lowPSMA_min_ and highPSMA_average_ (estimated median: 21.3 vs. 11.4 months; p = 0.02; HR = 0.3; 95%CI = 0.102-0.877; p = 0.028); patients classified as both lowPSMA_min_ and highPSMA_average_ had longer survival compared to lowPSMA_average_ (estimated median: 11.4 vs. 5.3 months; p = 0.002; HR = 0.364; 95%CI = 0.186-0.710; p = 0.003). Multivariate Regression (including binarized PSMA_min_ and lowPSMA_average_ and adjusted for presence of liver metastases) confirmed both highPSMA_min_ and highPSMA_average_ to be significant positive prognosticators of overall survival (highPSMA_average_: HR = 0.473; p = 0.044; 95%CI = 0.229-0.980 | highPSMA_min_: HR = 0.300; p = 0.028; 95%CI = 0.102-0.878 | liver metastases absence: HR = 0.476; p = 0.033; 95%CI = 0.240-0.943). Optimized threshold values (both absolute and relative to liver activity) for other SUV parameters are shown by *Table 5.* The median volumetric fraction of low PSMA expressing tumor volume was 3.6% (IQR: 13.8) for the proposed optimized log rank threshold (10.2 SUV). The volumetric fraction of low PSMA expressing tumor volume could significantly stratify the overall survival time (>9.7% vs. =<9.7%; p = 0.023; 6.4 vs. 11.4 months; only lowPSMA_min_ patients).

### Influence of liver metastases and Cabazitaxel therapy

There were no significant differences between patients with and without liver metastases regarding PSMA_min_ (9.1vs.8.9; p = 0.418) or PSMA_average_ (18.3 vs. 20.1, p = 0.264; *Figure 3*). The same was true for a positive/negative history of Cabazitaxel therapy (PSMA_min_ 8.9 vs. 9.1; p = 0.615; PSMA_average_ 15.9 vs. 20.1, p = 0.138; *Figure 3*). There were no statistically significant associations between the presence of liver metastases and PSMA_min_ status (p = 1.000) or PSMA_average_ status (p = 0.164). Likewise, there were no statistically significant associations between the history of Cabazitaxel therapy and PSMA_min_ status (p = 0.338) or PSMA_average_ status (p = 0.547). In accordance with a previous study of our group, presence of liver metastases (HR = 2.775; p = 0.001; 95%CI = 1.481-5.198) as well as history of second line chemotherapy (HR = 3.047; p = 0.002; 95%CI = 1.523-6.093) were significant negative prognosticators of overall survival 18.

## Discussion

The implication of low PSMA expression for the overall survival of patients treated with Lu-PSMA therapy was investigated in the present study. To this end, the correlation of overall survival and various PSMA-PET parameters was elucidated. Additionally, different PET uptake criteria have been employed to group patients into low or high PSMA expression. The highest pathological PSMA expression of a given patient under Lu-PSMA therapy (PSMA_max_) could not prognosticate overall survival. Low average PSMA expression of all metastases (PSMA_average_) was associated with shorter overall survival. The absence of low PSMA expressing metastases prior to Lu-PSMA therapy was associated with best overall survival. The volumetric fraction of low PSMA expressing metastases was a negative prognosticator of overall survival.

To date, there is no reasonable definition of low or high PSMA expression. However, patients in whom the PSMA expression was assessed low were not considered for Lu-PSMA therapy in the Australian Phase II trial of Hofman et al. 3,19. Yet, the benefit of the employed PSMA expression-based inclusion criteria could not be evaluated in the very same trial. Interestingly, some patients from the present cohort that received Lu-PSMA therapy had a lower maximum PSMA expression (SUV_max_ 7.1) compared to the Hofman et al. cohort (SUV_max_ 22.1) 3. Therefore, the present study retrospectively applied the PSMA-PET criterion of Hofman et al., to evaluate, if patients would have been judged eligible for Lu-PSMA therapy 3. In the present cohort, the maximum PSMA expression of each patient was above the liver specific threshold of Hofman et al. (1.5 × SUV_mean_ of liver) and therefore all would have met the inclusion criterion of Hofman et al. 3. Eight patients had at least one metastasis with a SUV_max_ below this liver specific threshold. Still, there was no significant stratification of patients according to overall survival in the present cohort. Finally, in the present patient cohort, PSMA_max_ was not a significant prognosticator of overall survival, which is in line with a recent publication of Ferdinandus et al. 20. Therefore, the maximum pathological PSMA expression seems unsuited to predict the therapy response of patients who show a minimum SUV_max_ of 7.1 at any metastatic site.

Patients with low average PSMA expression had short overall survival compared to those with high average PSMA expression and/or no low PSMA expressing metastases. Yet it remains unclear, if these patients still benefited from Lu-PSMA therapy. There is only limited evidence that the overall survival would have been worse, if Lu-PSMA therapy would not have been administered. The survival of patients with low PSMA expression that received Lu-PSMA therapy was longer in the current cohort (6.4 months) compared to patients excluded from the Lu-PSMA Phase II trial of Hofman et al. (2.5 months) 19. Other studies have employed the survival of historic control cohorts for comparison 14. But these comparisons might be heavily influenced by the individual metastatic spread and tumor differentiation, especially given the small patient numbers. Therefore, no rationale for PSMA-PET based exclusion criteria for Lu-PSMA can be provided by the given study. However, it seems unfavorable to use the maximum PSMA expression as decision criterion which did not correlate with overall survival.

Other prognosticators of Lu-PSMA therapy outcome have been identified in previous studies 18,21. Amongst others, the presence of liver metastases and history of second line Cabazitaxel chemotherapy were negative prognosticators of overall survival 18,22. Interestingly, there were no statistically significant associations between those predictors and the PSMA_average_ or PSMA_min_ status.

Due to genetic and non-genetic variations, cancer cells both in primary tumors and metastases become heterogeneous during the course of the disease 23,24. In prostate cancer, neuroendocrine differentiation of cancer cells may occur in advanced stages and especially after lasting androgen deprivation therapy 25-29. Prostate cancer cell markers like prostate-specific antigen are lost during dedifferentiation, whereas neuroendocrine markers like neurone specific enolase are gained 26,30. Neuroendocrine differentiation is generally associated with poor overall survival 31. Interestingly, Rathke et al. could show that the neuroendocrine marker chromogranin A is a moderately negative predictor for response in patients treated by Lu-PSMA therapy 12. In the present study, the average PSMA expression was a positive prognosticator for overall survival. Interestingly, average PSMA expression and variation of PSMA uptake (measured as PSMA_std_) between metastases were highly correlated. Both findings might be partly explained by dedifferentiation of prostate cancer cells in metastases. This might be contradictory, as one could assume that decreased average PSMA expression is associated with more heterogeneity between metastases (i.e. side by side presence of metastases with low and with high PSMA expression). However, it remains unclear if the shorter overall survival of patients with low PSMA expressing metastases is due to dedifferentiated and more aggressive tumor phenotypes, or due to reduced efficacy of Lu-PSMA therapy.

PSMA PET can visualize PSMA expression in the living patient and Lu-PSMA therapy targets PSMA expressing cells. Therefore, it has been hypothesized that PSMA PET can predict the achieved radiation dose, which is deposited in the tumor, and thereby indirectly predict the treatment response. The work of Violet et al. had shown that the mean whole body PSMA PET uptake indeed correlated with the absorbed doses of Lu-PSMA therapy 32. Moreover, patients that obtained doses <10 Gy had unfavorable PSA response rates 32. However, no survival data was studied by Violet et al. 32. In contrast, the present manuscript could show that mean whole body PSMA expression is a significant predictor of survival.

The overall survival of patients that present strong PSMA expressing metastases, but likewise have less PSMA expressing metastases is currently unclear. Interestingly, highPSMA_min_ patients had a significantly longer overall survival. Taken together with PSMA_average_, patients were stratified based on PSMA-PET measurements into those with low (lowPSMA_average_), intermediate (highPSMA_average_ and lowPSMA_min_) or high (highPSMA_min_) PSMA expression. Thereby, patients could be stratified in those with good, intermediate and poor overall survival. This is depicted by *Figure 2*. Moreover, the volumetric fraction of low PSMA expressing metastases was a negative prognosticator for overall survival. Future studies have to elucidate, if the occurrence of low PSMA expressing metastases (i.e. lowPSMA_min_) is sequentially followed by the tendency to PSMA decrease in all metastases (i.e. lowPSMA_average_) in the process of the disease. Moreover, it might be warranted to elucidate, if this sequence is caused by occurrence and spared of dedifferentiated tumor cell phenotypes.

The present study has limitations. It was conducted retrospectively and is therefore prone to selection biases. The patient collective might not be comparable to the Phase II trial of Hofman et al. or other retrospective analyses 13. However, retrospective studies are mandatory for the planning of prospective trials, especially for inclusion criteria definition. In the present analysis, no FDG PET-CT was employed for patient selection. Therefore, PSMA negative metastases that show strong FDG uptake might have evaded the analysis. Additionally, small metastases (<0.5 ml) were no considered in the analysis. The SUV_mean_ heavily depends on the segmentation method. In contrast to previous approaches, we did not use a fixed SUV threshold (e.g. SUV > 3) but relative thresholding (50% of local SUV_max_). Therefore, SUV_mean_ results are not directly comparable with other studies. Because of that, only SUV_max_ based PET parameters (PSMA_max_, PSMA_average_, PSMA_min_, PSMA_std_) were used for hypotheses generation.

## Conclusion

In this retrospective analysis, PSMA_average_ was a significant prognosticator for overall survival of mCRPC patients treated with Lu-PSMA therapy, whereas PSMA_max_ was not. Patients without low PSMA expressing metastases had the best overall survival. Future studies are warranted to elucidate the degree of low PSMA expression tolerable for Lu-PSMA therapy.

## Figures and Tables

**Table 1:** Patient characteristics

Patient characteristics	N [%]	Median [IQR]; survival: Median [CI]
Number of patients	85 [100%]	
Age (years)		73.1 [11.4]
Estimated overall survival time (months)		11.4 [8.0-14.7]
>50% PSA decline from baseline	39 [46%]; n = 80, follow up not present for 5 patients.	
**PSMA therapy**		
Number of cycles		3.0 4
Cumulated activity (GBq)		19.3 [24.8]
**Baseline blood parameters**		
Alkaline phosphatase (U/l)		147.0 [193.0]
Lactate dehydrogenase (U/l)		316.5 [227.0]
Aspartate aminotransferase (U/l)		32.5 [24.0]
Alanine transaminase (U/l)		16.0 [11.0]
Hemoglobin (g/dl)		10.4 [2.4]
Prostate-specific antigen (ng/ml)		284.0 [805.0]
**Metastases**		
Bone	78 [92%]	
Lymph node	68 [80%]	
Liver	26 [31%]	
Lung	20 [24%]	
Brain	1 [1%]	
**Previous therapies**		
Docetaxel	68 [80%]	
Cabazitaxel	20 [24%]	
Abiraterone	72 [85%]	
Enzalutamide	72 [85%]	

Blood parameters were not available for all patients; Abbreviations: Std = standard deviation; CI = confidence interval.

**Table 2:** SUV parameters of the presented patient cohort (n = 85)

PET parameter	Median of the *average* value of all patients	Median of the *highest* value of all patients	Median of the *lowest* value of all patients
SUVmax	18.9 [5.9-73.4]	44.6 [7.1-181.6]	8.9 [2.7-40.9]
SUVmean	13.0 [4.0-51.4]	29.5 [4.6-129.8]	6.3 [2.6-29.4]
SUVpeak	12.1 [3.7-46.5]	31.6 [4.7-159.7]	5.7 [1.8-22.6]

Abbreviation: Squared brackets = range.

**Table 3:** Baseline PSMA PET parameters and overall survival

	Measurement selected per patient	HR	95%CI	P
SUV_max_	Average (PSMA_average_)	0.959	0.921-0.999	**0.047**
	Highest (PSMA_max_)	0.992	0.979-1.005	0.231
	Lowest (PSMA_min_)	0.890	0.768-1.030	0.118
	Std (PSMA_std_)	0.936	0.877-0.999	**0.048**
SUV_max_ / SUV_mean liver_	Average	0.963	0.895-1.036	0.313
	Highest	0.996	0.975-1.017	0.701
	Lowest	0.904	0.728-1.123	0.363
	Std	0.932	0.822-1.057	0.274
SUV_mean_	Average	0.941	0.887-0.999	**0.045**
	Highest	0.989	0.970-1.008	0.241
	Lowest	0.799	0.638-1.002	0.052
	Std	0.904	0.820-0.996	**0.042**
SUV_peak_	Average	0.941	0.882-1.004	0.064
	Highest	0.989	0.972-1.007	0.227
	Lowest	0.736	0.533-1.016	0.062
	Std	0.918	0.844-0.999	**0.048**

Abbreviations: HR = Hazard ratio; CI = confidence interval; Std = standard deviation.

**Table 4:** Overlap of PET stratification

	highPSMA_average_	lowPSMA_average_	Sum
lowPSMA_min_	49	20	*69*
highPSMA_min_	16	0	*16*
**Sum**	*65*	*20*	*85*

Low/highPSMA_min_ = patients with or without metastases that had a SUV_max_ above or below 10.2; high/lowPSMA_average_ = patients with an average SUV_max_ of all metastases above or below 14.3. SUV threshold values resemble optimized log rank cut-offs.

**Table 5:** Binarized baseline PSMA PET parameters and overall survival

	Measurement selected per patient	Ideal cut-off	P	Below cut-off			Above cut-off		
				n	Median Survival	95%CI	n	Median Survival	95%CI
SUV_max_	*Average (=PSMA_average_)*	14.3	**<0.001**	20	5.3	2.3-8.8	65	15.1	7.8-23.4
	*Highest (=PSMA_max_)*	n.a.	n.s.						
	*Lowest (=PSMA_min_)*	10.2	**0.003**	69	7.9	4.6-11.2	16	21.3	n.a.
	*Std (=PSMA_std_)*	3.0	**<0.001**	9	3.2	0.0-6.9	76	13.2	9.0-17.4
SUV_max_ / SUV_mean liver_	*Average*	6.2	**0.003**	42	7.2	5.8-8.6	43	21.3	13.2-29.3
	*Highest*	n.a.	n.s.						
	*Lowest*	n.a.	n.s.						
	*Std*	n.a.	n.s.						
SUV_mean_	*Average*	9.3	**<0.001**	17	6.4	1.7-11.1	68	15.1	10.1-20.1
	*Highest*	n.a.	n.s.						
	*Lowest*	7.1	**0.004**	65	8.6	4.3-12.9	20	25.5	15.1-36.0
	*Std*	1.9	**<0.001**	9	3.2	0.0-6.9	76	13.2	9.1-17.4
SUV_peak_	*Average*	9.7	**<0.001**	19	6.4	3.7-9.1	66	13.2	8.4-18.1
	*Highest*	n.a.	n.s.						
	*Lowest*	6.6	**0.004**	65	8.6	4.3-12.9	20	25.5	15.1-36.0
	*Std*	n.a.	n.s.						

Abbreviations: CI = confidence interval; Std = standard deviation; n.a. = not available; n.s. = not statistically significant. Bonferroni adjustment: p < 0.0125 is regarded statistically significant. Ideal cut-off was found by log rank cut-off finder.

## Abbreviations

PET: Positron Emission Tomography
PSMA: Prostate Specific Membrane Antigen
